# Differing responses of osteogenic cell lines to β-glycerophosphate

**DOI:** 10.1038/s41598-023-40835-w

**Published:** 2023-09-02

**Authors:** Olga S. Yevlashevskaya, Ben A. Scheven, A. Damien Walmsley, Richard M. Shelton

**Affiliations:** School of Dentistry, College of Medical and Dental Sciences, University of Birmingham, 5 Mill Pool Way, Edgbaston, Birmingham, B5 7EG UK

**Keywords:** Stem-cell differentiation, Cell division

## Abstract

Ascorbic acid (Asc), dexamethasone (Dex) and β-glycerophosphate (β-Gly) are commonly used to promote osteogenic behaviour by osteoblasts in vitro. According to the literature, several osteosarcoma cells lines appear to respond differently to the latter with regards to proliferation kinetics and osteogenic gene transcription. Unsurprisingly, these differences lead to contrasting data between publications that necessitate preliminary studies to confirm the phenotype of the chosen osteosarcoma cell line in the presence of Asc, Dex and β-Gly. The present study exposed Saos-2 cells to different combinations of Asc, Dex and β-Gly for 14 days and compared the response with immortalised human mesenchymal stromal/stem cells (MSCs). Cell numbers, cytotoxicity, mineralised matrix deposition and cell proliferation were analysed to assess osteoblast-like behaviour in the presence of Asc, Dex and β-Gly. Additionally, gene expression of runt-related transcription factor 2 (*RUNX2*); osteocalcin (*OCN*); alkaline phosphatase (*ALP*); phosphate regulating endopeptidase homolog X-linked (*PHEX*); marker of proliferation *MKI67* and proliferating cell nuclear antigen (*PCNA*) was performed every two days during the 14-day cultures. It was found that proliferation of Saos-2 cells was significantly decreased by the presence of β-Gly which contrasted with hMSCs where no change was observed. Furthermore, unlike hMSCs, Saos-2 cells demonstrated an upregulated expression of late osteoblastic markers, *OCN* and *PHEX* that suggested β-Gly could affect later stages of osteogenic differentiation. In summary, it is important to consider that β-Gly significantly affects key cell processes of Saos-2 when using it as an osteoblast-like cell model.

## Introduction

Osteogenic supplementation consisting of ascorbic acid (Asc), dexamethasone (Dex) and β-glycerophosphate (β-Gly) is routinely used to induce the osteogenic phenotype in osteoblast cultures^1,2^ Asc serves as a cofactor for proline hydroxylation involved in collagen synthesis, which is required for mineral matrix deposition^3^, whilst Dex promotes osteogenic differentiation by upregulating the transcription of *RUNX2*^1,4^. β-Gly provides a source of organic phosphate for extracellular matrix mineralisation in vitro^5^.

However, with the variety of osteogenic models available including the range of osteosarcoma cell lines MG-63, U2OS and Saos-2, the reported effects of Asc, Dex and β-Gly in vitro are not consistent in the literature. Valenti et al.^6^ showed Asc-induced osteogenic differentiation at up to 250 µM with no signs of apoptosis below 750 µM in relatively undifferentiated osteoblast-like MG-63 cells. Whereas Cmoch et al.^7^ described activated matrix mineralisation accompanied by apoptosis in more fully differentiated Saos-2 cells exposed to 283 µM Asc and 7.5 mM β-Gly. The latter suggested a possible role of β-Gly in apoptosis which was not addressed by the authors. Orimo and Shimada^8^ used 10 mM β-Gly in Saos-2 cells and reported reduced cell numbers which was suggested to be linked to apoptosis. Additionally, there was an upregulated expression of *PHEX* and *MEPE*^9^ both associated with decreased mineral production and differentiation into osteocytes. In contrast, Cmoch et al.^7^ observed increased mineralisation by Saos-2 cells at a similar β-Gly concentration. However, whilst many studies use β-Gly, some authors recommend avoiding the use of ~ 10 mM β-Gly as it causes non-physiological levels of extracellular phosphate that can alter normal osteogenic gene transcription^10^. Moreover, there have been conflicting reports on the influence of Dex in osteogenic induction in vitro. Coelho and Fernandes^11^ reported significantly higher cell numbers in human mesenchymal stromal/stem cells (MSCs) exposed to 10^–8^ M Dex than in controls. This contrasted with Sordi et al.^12^ using 10 mM Dex for osteogenic induction where a decrease in proliferation of human exfoliated deciduous teeth cells was observed. Some studies^7^ omitted the use of Dex in osteogenic stimulation completely and still observed matrix mineralisation.

Osteosarcoma is an heterogenous cancer and the cell lines derived from such tumours show varying phenotypes^13^. Thus, the choice of osteosarcoma cell line as an osteoblast-like model can significantly affect the outcome of in vitro experiments. The role of Asc, Dex and β-Gly in key cell processes including proliferation, apoptosis and osteogenic gene expression must be defined specifically for particular osteosarcoma cell lines to produce data which is comparable. The present study compared the response of the Saos-2 osteosarcoma cell line and a non-carcinogenic cell model with Asc, Dex and β-Gly. Saos-2 is a commonly used osteoblast-like cell line derived from an 11-year-old Caucasian female patient suffering from primary osteosarcoma^14^. Saos-2 presents features characteristic of relatively mature osteoblasts including mineralised matrix synthesis, expression of 1,25-dihydroxyvitamin D3 receptors and elevated production of collagen-1^3^ Furthermore, the relative ease of maintenance of Saos-2 in tissue culture and the short doubling time make it an attractive cell model for in vitro research. However, the cancerous nature of the Saos-2 cells causes several biological variations including altered proliferation kinetics and the absence of contact inhibition in vitro^15^. The latter contrasts markedly with the in vivo environment and therefore limits the physiological relevance of data obtained using these cells so a non-tumour derived cell model was used to compare the effects of Asc, Dex and β-Gly with those from Saos-2. An immortalised human bone marrow cell line (hMSC) was selected due to its non-cancerous origin in contrast with Saos-2. The hMSC line was immortalised via serial passaging combined with viral-mediated transfection of SV40 large T antigen^16^. However, despite a shorter doubling time than observed in primary cells, the hMSC line retains contact inhibition properties and the potential for adipogenic, chondrogenic and osteogenic differentiation. As a result, the hMSC line offers a physiologically relevant osteoblast-like model system while still allowing for the convenience of working with a cell line.

Considering the conflicting data discussed, the aim of the present study was to compare the effect of Asc, Dex and β-Gly on the osteogenic behaviour of the Saos-2 cell line and a relatively undifferentiated non-cancer derived hMSC line. This investigation provides a unique set of data demonstrating the relevance of Saos-2 as an osteoblast-like model system in vitro.

## Materials and methods

### Cell culture and osteogenic differentiation

Saos-2 and non-differentiated hMSCs were obtained from laboratory stores and Abm (T0520, Abm, Canada) respectively. Saos-2 cells were maintained in Dulbecco’s Modified Eagle’s Medium (DMEM)/Ham’s Nutrient Mixture F12 (Sigma, UK) supplemented with 1% penicillin/streptomycin, 1% l-glutamine and 10% foetal bovine serum (FBS). hMSCs were cultured in DMEM (low glucose, pyruvate) (Gibco, UK) with 0.5% penicillin/streptomycin and 10% FBS. Both cell lines were maintained at 37 °C in an atmosphere of 5% CO_2_. Cells were seeded at a density of 8.4 × 10^3^ cells cm^−2^ and expanded for 72 h until confluence which was day 0 of the experiment. Osteogenic supplements were introduced on day 0 using 283 µM Asc, 9.3 mM β-Gly and 10^–8^ M Dex. The following combinations of osteogenic supplements were established: control (no osteogenic supplementation); Asc Dex β-Gly; Asc Dex; Asc β-Gly; Dex β-Gly; Asc; Dex and β-Gly. Cells were incubated in variously supplemented culture media for up to 14 days with a change of media every 3 days. Cell growth in terms of numbers was examined every 2 days until day 14 of incubation. First, cell monolayers were detached from the polystyrene surface using 0.05% trypsin in 0.02% EDTA (Sigma, UK) for 10 min at 37 °C, centrifuged and resuspended in 1 ml 10% FBS in DMEM/Ham’s F-12. Viable cell counts were performed using trypan blue staining and a Neubauer haemocytometer.

### Cytotoxicity assay

The lactate dehydrogenase (LDH) cytotoxicity assay was performed to examine cell death in cultures by quantifying the release of LDH. The LDH-Glo™ kit was used (Promega, UK) according to the manufacturer’s instructions. Briefly, a 2 µl aliquot of culture media was collected on day 7 of incubation in osteogenic media and diluted in 50 µl LDH storage buffer before 25 µl of this solution was transferred into an opaque walled, transparent bottom 96 well plate and mixed with an equal volume of LDH detection reagent. Luminescence was recorded using a plate reader (Tecan Spark, Switzerland) after 60 min of incubation at room temperature. Two negative controls were used in this experiment: a no cells control—to assess the background luminescence of the culture media and no treatment control—to assess cell death in cultures without the osteogenic supplementation. A positive control with LDH was prepared by permeabilising the no treatment control cells with 10% Triton-X100 for 10 min immediately before collecting the culture media samples.

### Alizarin red S staining

To examine mineral deposition in cell cultures Alizarin red S staining (ARS) was performed on day 14 of incubation in osteogenic media as described by Gregory et al.^17^. The cells were fixed with 10% formalin and stained with 40 mM Alizarin red S stain (pH 4.2) for 20 min at room temperature. Stain quantification was performed by extracting the stain with 10% acetic acid for 30 min followed by neutralisation with 0.1% ammonium hydroxide. The optical density of samples was quantified using a plate reader at 450 nm and compared with a standard curve of serial dilutions.

### Assessment of gene expression using qPCR

Total RNA was isolated every 2 days up to day 14 of osteogenic treatment using the RNeasy Mini kit (Qiagen, UK) according to the manufacturer’s instructions. The samples were generated in 3 separate experiments to produce 3 biological replicates. Cell monolayers were dissociated using a lysis buffer followed by dilution with the 70% molecular grade ethanol. Then the suspensions were passed through the RNeasy spin column and centrifuged at 10,000 RPM for 30 s. Any non-specifically bound organic molecules and salts were removed using a wash buffer provided in the kit. Genomic DNA was digested using the On-Column DNAse kit (Sigma, UK) by adding the DNase solution directly to the samples and incubating for 15 min at room temperature. RNA was collected by adding 30 µl of RNAse-free water to the samples and centrifuging at 10,000 RPM for 1 min before storing the RNA samples at − 80 °C until later use. The quality of the resulting RNA was established using spectrophotometry (Genova Nano, Jenway™, UK).

Then, complementary DNA (cDNA) was produced from the isolated RNA via reverse transcription (RT) using the Tetro cDNA synthesis kit (Bioline, UK). The reaction master mix was prepared by combining 1 µl of 10 mM deoxynulceoside triphosphate (dNTP) mix, 4 µl of RT reaction buffer, 1 µl of RNase inhibitor and 1 µl of reverse transcriptase (200 U/µl). The reaction contained 8 µl of the master mix, 2 µg of RNA sample and DEPC-treated water to a final reaction volume of 20 µl. The mix was transferred to the thermocycler (Veriti, 96 well thermal cycler, Applied Biosystems, USA) and incubated at 45 °C for 1 h to allow RT followed by 85 °C for 5 min to terminate the reaction. The resulting cDNA samples were stored at − 20 °C before use.

Finally, real-time PCR (qPCR) was performed using LightCycler® 480 SYBR Green I Master and 480 LightCycler® system (Roche Diagnostics). The expression of the following osteogenic markers was analysed: runt-related transcription factor 2 (*RUNX2*); osteocalcin (*OCN*); alkaline phosphatase (*ALP*) and phosphate regulating endopeptidase homolog X-linked (*PHEX*). The expression of a marker of proliferation (*MKI67*) and proliferating cell nuclear antigen (*PCNA*) were used to provide a comparison of proliferative markers. Sequences of the primers used are provided in supplementary materials. The gene expression levels were normalised to the expression of *YWHAZ* (tyrosine 3-monooxygenase). Data quantification was performed using the second derivative maximum method. This involved an algorithm identifying crossing points (Cp)—the number of PCR cycles after which the exponential phase of the target sequence amplification began. The starting concentration of target gene samples were found by comparing the Cp values against those in the standard curve of serial dilutions of log primer concentrations.

### Determination of cell proliferation

Cell proliferation was examined on day 4 of incubation with full osteogenic culture media (Asc, Dex and β-Gly) to avoid examining confluent cell layers where proliferation may be compromised by contact inhibition. Cultures were incubated in 6 well plates. BrdU (5-Bromo-2′-deoxy-uridine) uptake was measured to identify replicating cells according to the manufacturer’s instructions (ab287841, Abcam, UK). Briefly, cells were exposed to the BrdU labelling solution for 6 h at 37 °C in 5% CO_2_. Samples were fixed with ethanol (50 mM glycine, 70% ethanol) for 20 min at − 20 °C and washed three times with the washing buffer provided in the kit. Then, samples were incubated with anti-BrdU antibody for 30 min at 37 °C and washed again, followed by incubation with anti-mouse Ig-fluorescein antibody for 30 min at 37 °C. Mounting media containing DAPI was applied for 10 min at room temperature to stain cell nuclei. BrdU and DAPI incorporation were examined using fluorescence microscopy (Eclipse TE300, Nikon, Japan). The following negative controls were established to prevent non-specific staining: (1) no BrdU, (2) no anti-BrdU antibody, (3) no secondary antibody. Images were captures using digital camera (D5100, Nikon, Japan). Brightness and contrast were enhanced equally in all images using Image J without obscuring or eliminating any details. BrdU-stained and non-stained nuclei were counted manually.

### Statistical analysis

All experiments were established with three technical replicates (i.e., 3 wells) and undertaken independently 3 times. The data was presented as the median of 3 biological replicates as well as the minimum, maximum, lower quartile, and upper quartile values. Differences in parametric data were compared using Brown-Forthyse and Welch ANOVA tests and P < 0.05 was considered statistically significant. Statistical analysis and figures were generated using GraphPad Prism, Version 9.5.0 (GraphPad Software, San Diego, California US).

## Results

### Cell growth

**Figure 1:**
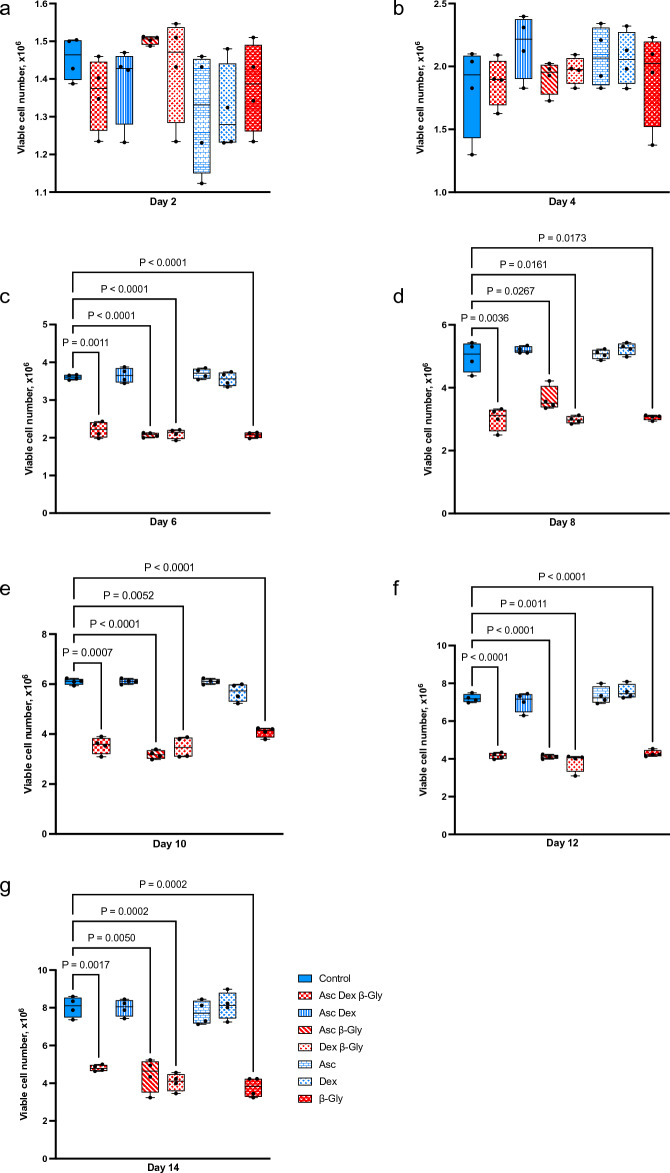
Mean viable cell numbers of Saos-2 after incubation with different combinations of osteogenic supplements for 14 days. Numbers of Saos-2 cells were significantly reduced in the presence of β-Gly after 4 days of supplementation relative to the control of the corresponding day (**c**–**g**). Asc and/or Dex did not affect Saos-2 numbers compared with the control (**a**–**g**). Blue and red bars represent cultures without and with β-Gly respectively. n = 3.

**Figure 2:**
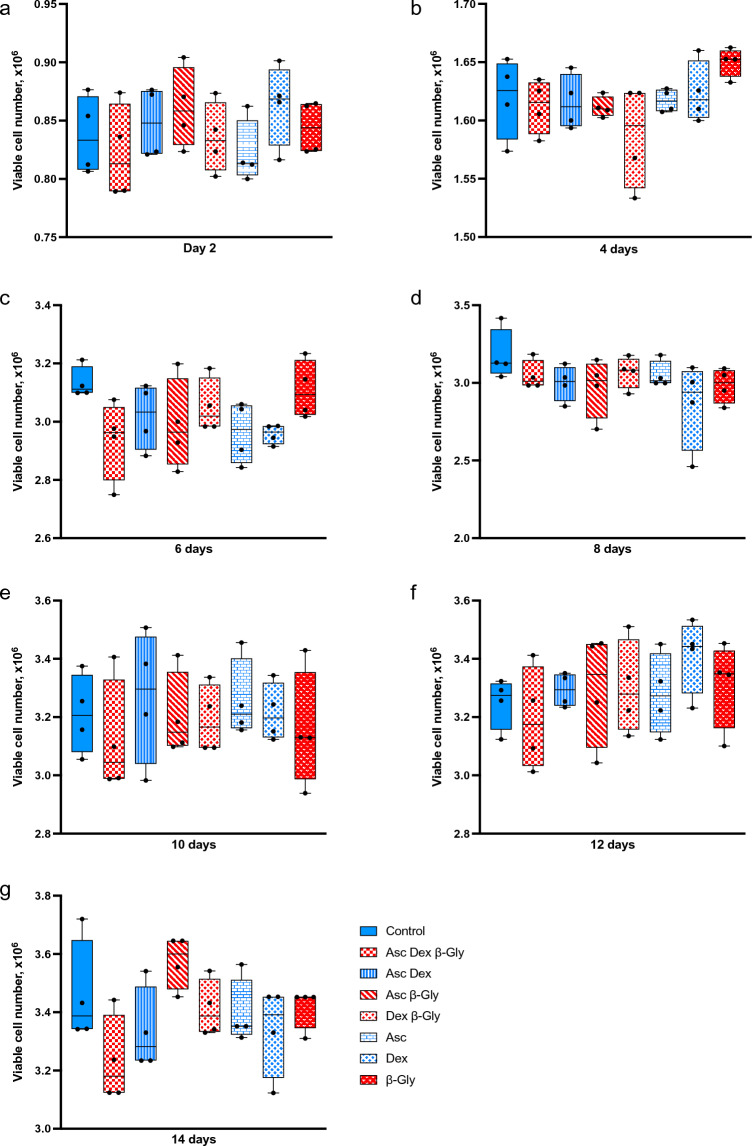
Mean viable cell numbers of hMSCs after incubation with different combinations of osteogenic supplements for 14 days. In contrast to the Saos-2 cells (Fig. 1), hMSCs did not show a significant change in numbers with the addition of β-Gly. Nor did Asc and/or Dex affect hMSCs numbers compared with the control. Blue and red bars represent cultures without and with β-Gly respectively. n = 3.

The effect of osteogenic supplements on cell growth was studied by quantifying viable cell numbers for 14 days. Saos-2 cultures exposed to β-Gly demonstrated a significantly lower number of viable cells compared with Dex and/or Asc supplemented cultures after day 4 of incubation (Fig. 1) and at all subsequent time points examined. In contrast, the number of viable hMSCs was not statistically significantly affected by any of the osteogenic supplements in comparison with controls (Fig. 2).

### Cytotoxicity assay

**Figure 3:**
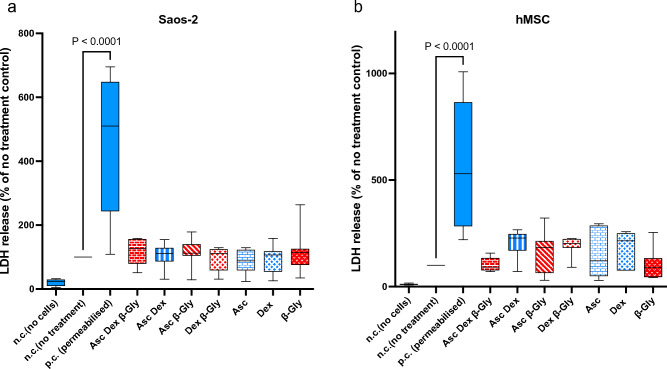
The LDH assay demonstrates LDH released from damaged/dead cells. A negligible amount of LDH was detected in the no-cells negative control (n.c.). A 4.5-fold increase in LDH was observed in the positive control (p.c.) containing Triton-permeabilised cells when compared with no treatment controls. No significant change in LDH release was seen between the different osteogenic supplemented cultures in either Saos-2 (**a**) or hMSCs (**b**). Red bars represent cultures with β-Gly and blue bars do not. n = 3.

To evaluate the cause of the reduced Saos-2 cell numbers in β-Gly-positive cultures an LDH assay was performed. No significant change in LDH release was observed between the osteogenically treated cells and controls (Fig. 3) in either Saos-2 (A) or hMCSs (B).

### Proliferation assay

**Figure 4:**
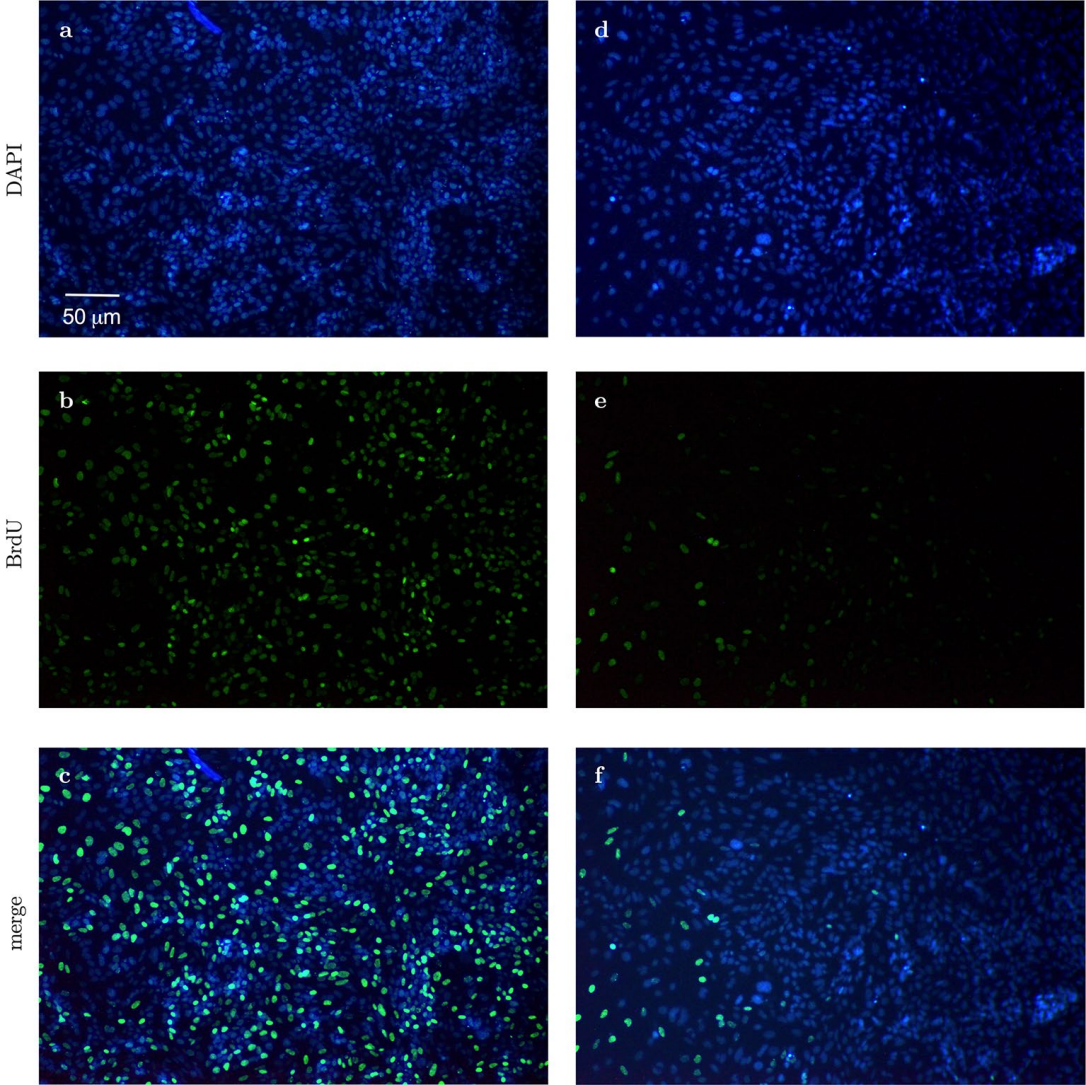
Representative fluorescence micrographs of BrdU staining of Saos-2 cells after 4 days supplementation with β-Gly. Saos-2 cells exposed to β-Gly (**d**–**f**) demonstrated a lower proportion of BrdU-stained nuclei than Saos-2 controls (**a**–**c**). n = 3.

**Figure 5:**
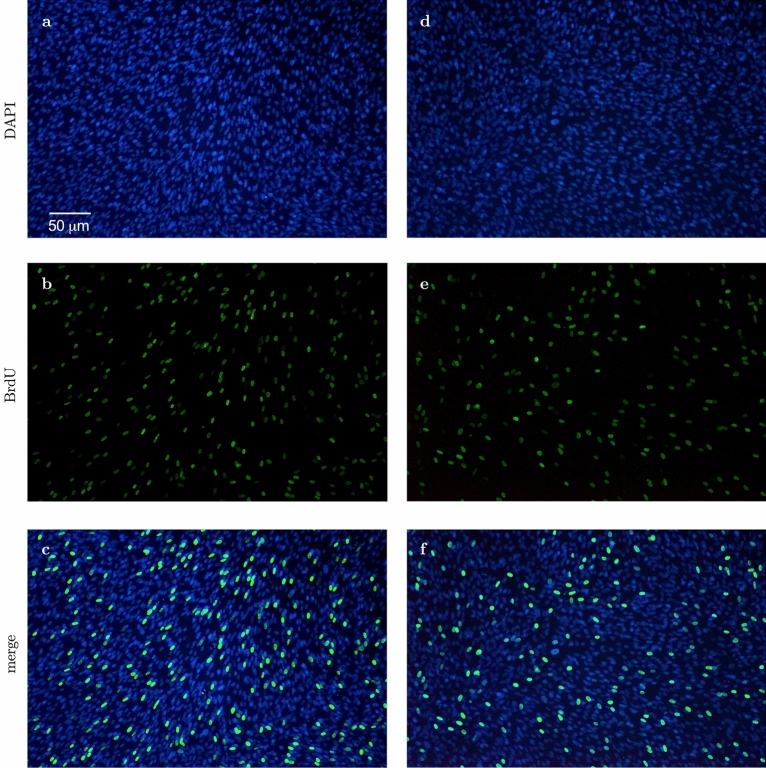
Representative fluorescence micrographs of BrdU staining of hMSCs after 4 days supplementation with β-Gly. The proportion of BrdU-labelled hMSCs nuclei did not differ significantly between β-Gly-positive (**d**–**f**) and β-Gly-negative (**a**–**c**) cultures. n = 3.

**Figure 6:**
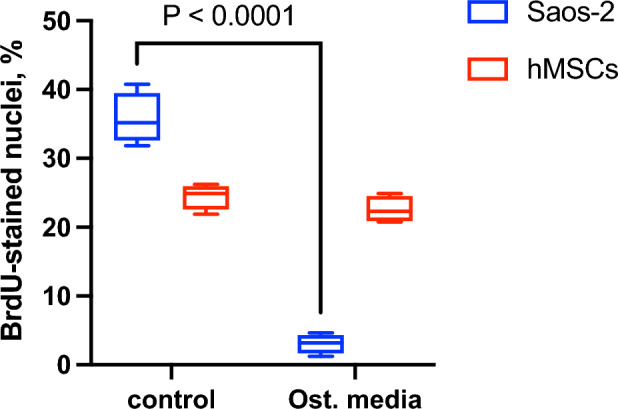
Saos-2 exposed to β-Gly demonstrated a lower proportion of BrdU-stained nuclei than Saos-2 controls, 3.1% and 35.6% respectively. β-Gly-positive and β-Gly-negative hMSCs cultures did not show a significant difference in the proportion of BrdU labelled nuclei—23.7% and 25% respectively. n = 3.

Saos-2 cells incubated in the presence of β-Gly showed significantly fewer (3.1%) BrdU-stained nuclei than cells not exposed to β-Gly (Figs. 4, 6) with 35.6% of all nuclei being stained with BrdU. Hence, the proliferative activity of Saos-2 was decreased in the presence of β-Gly. No statistically significant difference was identified in the number of BrdU-stained nuclei in hMSCs with 23.7% of BrdU stained nuclei in β-Gly exposed cells and 25% of BrdU-stained nuclei in negative controls (Figs. 5, 6). Therefore, cell proliferation was not affected by the presence of β-Gly in hMSCs relative to control cultures. Negative controls with either no BrdU, no anti-BrdU antibody or no secondary antibody did not generate any fluorescence.

### Alizarin red staining

**Figure 7:**
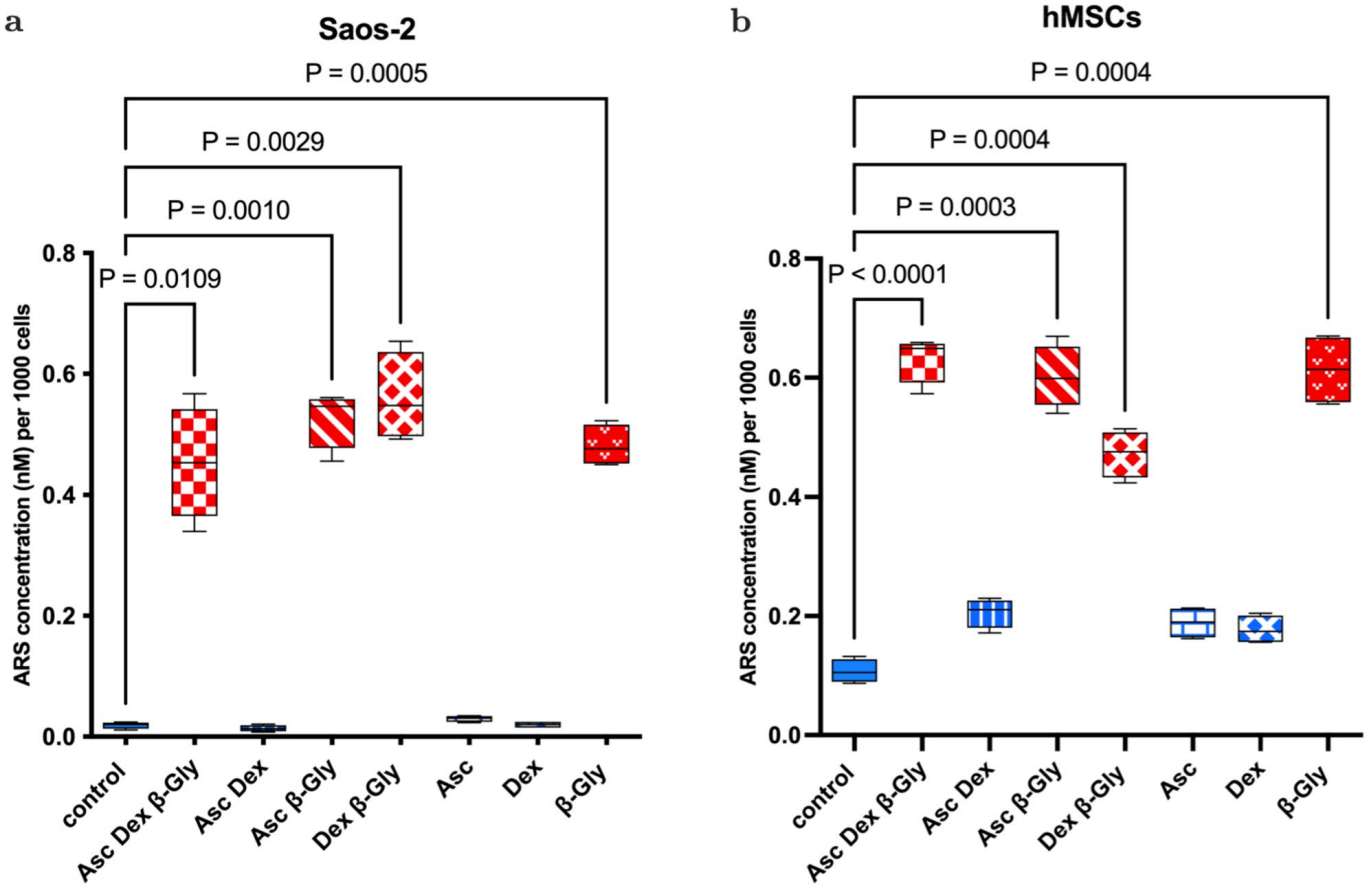
ARS showed a significant increase in calcified matrix synthesis in the presence of β-Gly in Saos-2 (**a**) and hMSCs (**b**) cultures. Asc and Dex did not increase mineral matrix production in either cell line. Red bars represent cultures with β-Gly and blue bars do not. n = 3.

ARS was used to assess mineral matrix deposition in osteogenically-supplemented cultures on day 14 of incubation. ARS showed a 24 and four-fold increase in mineral matrix production in β-Gly supplemented Saos-2 and hMSC cultures respectively (Fig. 7). Asc and Dex did not appear to affect mineral deposition in either cell line. However, there was an increased ARS concentration in β-Gly-negative hMSCs compared with in β-Gly-negative Saos-2.

### Gene expression analysis

**Figure 8:**
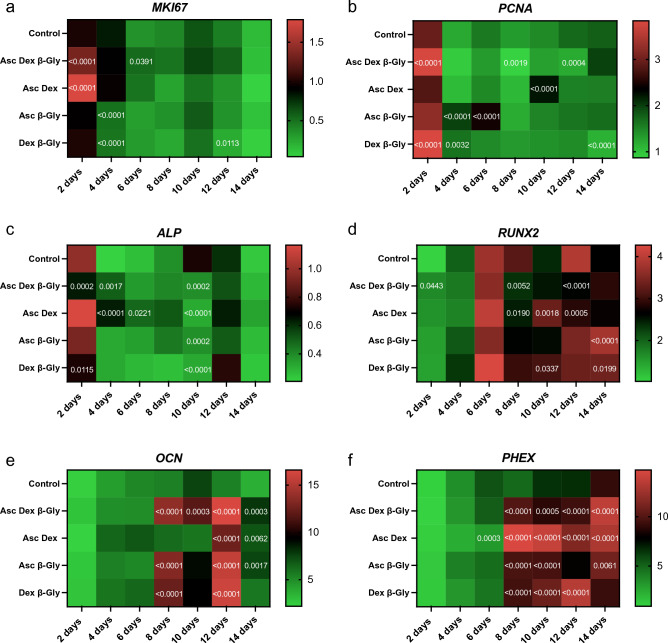
Heatmaps for the relative gene expression levels by Saos-2 cells incubated in osteogenic media over 14 days compared with the expression of *YWHAZ*. The data from each osteogenic condition were statistically compared with the control cultures at the corresponding time point. Where significant statistical differences existed, these are indicated as P values. The expression of proliferation markers *MKI67* (**a**) and *PCNA* (**b**) decreased over 14 days in all conditions. The activity concentration of *ALP* mRNA decreased (**c**) followed by significantly upregulated *RUNX2* expression (**d**) as of day 4 in the presence of β-Gly. A later osteoblast marker, *OCN* and the osteocyte-characteristic marker, *PHEX* increased expression after 8 days in β-Gly supplemented Saos-2 (**e**, **f**). n = 3. The heatmaps were created using GraphPad Prism, Version 9.5.0 (https://www.graphpad.com/updates/prism-950-release-notes).

**Figure 9:**
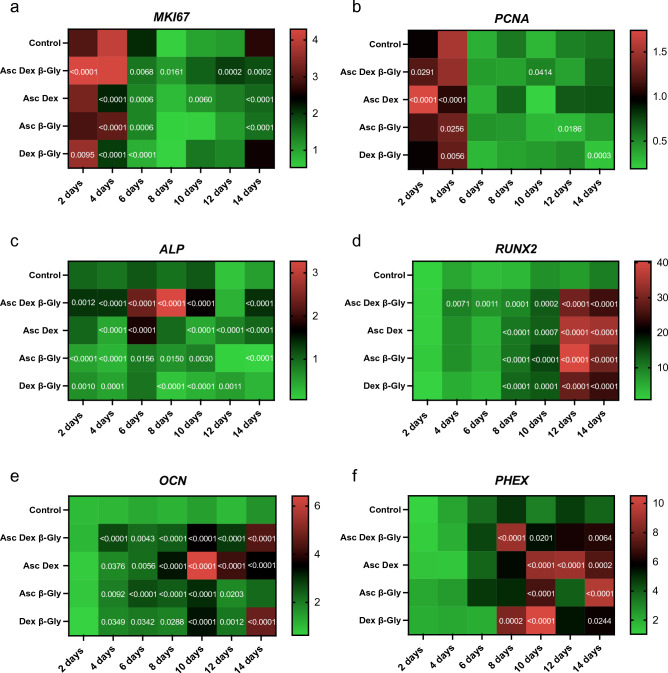
Heatmaps for the gene expression levels by hMSCs incubated in osteogenic media over 14 days relative to the expression of *YWHAZ*. The data from each osteogenic condition were statistically analysed in comparison with control cultures at the corresponding time point. Where significant statistical differences existed, these are indicated as P values. Like Saos-2 cells, hMSCs showed downregulation in the expression of *MKI67* (**a**) and *PCNA* (**b**) for all culture conditions. *ALP* (**c**) expression was upregulated in all osteogenic conditions, with the highest increase observed with Dex, Asc, and β-Gly. The upregulation peaked on day 8 of incubation*. RUNX2* expression increased only in hMSCs after 8 days of incubation (**d**). A less marked increase in transcription of *OCN* and *PHEX* was detected in β-Gly supplemented hMSCs after 8 days (**e**, **f**) than in Saos-2. n = 3. The heatmaps were created using GraphPad Prism, Version 9.5.0 (https://www.graphpad.com/updates/prism-950-release-notes).

The expression of proliferation markers and osteoblast-associated genes was normalised to the expression of *YWHAZ* and determined every two days of incubation with the different combinations of osteogenic supplements (Figs. 8, 9). Saos-2 cells demonstrated a gradual decrease in *MKI67* (Fig. 8a) and *PCNA* (Fig. 8b) expression during the 14 days, however a clear difference between cells exposed to different combinations of Asc, Dex and β-Gly was not identified. hMSCs showed a similar trend for *MKI67* expression (Fig. 9a) but a more marked decrease in the mRNA synthesis of *PCNA* (Fig. 9b) after 4 days of osteogenic supplementation than Saos-2 (Fig. 8b). *RUNX2* expression was significantly upregulated by the presence of osteogenic supplements in Saos-2 cells and increased during the incubation period relative to negative controls (Fig. 8d). In contrast, the *RUNX2* activity increased only at day 8 of the experiment and thereafter in osteogenically supplemented hMSCs (Fig. 9d). *ALP* expression was upregulated in the Dex, Asc, β-Gly supplemented hMSCs (Fig. 9c) and peaked on day 8 of incubation. Saos-2 showed an upregulation of *OCN* in the presence of β-Gly (Fig. 8e) at day 8 of the experiment. *PHEX*, an osteocyte-associated gene was significantly activated in all Saos-2 cultures containing osteogenic supplements at day 8 of incubation (Fig. 8f). No significant trend in *PHEX* expression was observed in hMSCs (Fig. 9f) at any stage of the experiment.

## Discussion

The diverse in vitro models used in osteoblast research create challenges with comparability of data between studies^14,15^. Osteosarcoma cell lines represent an affordable and straightforward option for researchers, however different cell origins can generate varying osteogenic gene expression profiles and phenotypes. The latter can produce variable data between studies and influence potential interpretation. The present study evaluated the response of an osteosarcoma cell line, Saos-2 to Asc, Dex and β-Gly used to induce osteogenic cell behaviour. An immortalised multipotent hMSC line was used for comparison of responses to osteogenic supplements by Saos-2 that may have been influenced by its tumour origin. It is crucial to emphasize that although the hMSCs did not originate from malignant tissue, they were immortalized through telomerase induction using transformation with SV40 Large T antigen^16^. This process resulted in a cellular phenotype that is more physiologically representative and "normal" when compared with Saos-2 cells. However, it is important to acknowledge that the cell line underwent certain modifications to achieve this state.

Previously it has been reported that^6,7^ Asc and/or Dex supplementation did not affect cell numbers in osteosarcoma cell lines including MG63 and Saos-3 suggesting either an anti-proliferative or toxic effect of β-Gly. However, in the present study Saos-2 cells demonstrated a decrease in cell number in the presence of β-Gly (Fig. 1) which was not observed in hMSCs (Fig. 2). β-Gly is used to promote mineralisation of the ECM by providing organic phosphate ions^5^. Inorganic phosphate concentrations of 5–7 mM in culture medium have been reported to induce apoptosis in osteoblasts^18^, possibly mediated by the mitochondrial damage via hyperpolarisation of the electrochemical gradient across the inner mitochondrial membrane and a consequent release of excess reactive oxygen species^19,20^ However, it has also been indicated that organic phosphates, such as β-Gly were not harmful to cells at similar concentrations^21^. Furthermore, osteoblasts are suggested to be adapted to the elevated phosphate concentrations in comparison with other cell types arising from involvement in bone-remodelling^18^. In the present study, the LDH assay confirmed that β-Gly did not change cell viability and therefore was not cytotoxic to Saos-2 at 9.3 mM (Fig. 3).

Interestingly, β-Gly appeared to decrease proliferation of Saos-2 cells but not hMSCs as shown by the BrdU staining (Figs. 4, 5 and 6). A decrease in cell proliferation rate and arrest in the G_0_ phase are expected events at the later stages of the cell cycle^22^, which would explain the decrease in cell numbers in the later phases of incubation of Saos-2 cells. Mature cell phenotypes can enter a non-dividing, G_0_ state via either terminal differentiation, senescence or quiescence^23^, expressing low to non-detectable levels of *MKI67* and *PCNA* mRNA^24^ *MKI67* gene codes for Ki-67, a protein involved in the formation of the perichromosomal layer necessary for chromosome condensation in mitosis^25^. It is expressed in G_1_, S, G_2_ and M phases of the cell cycle^26^. Whereas *PCNA*, typically transcribed in G_1_ and S^27^, codes for an accessory protein for DNA polymerase alpha^26^. Hence, the increased activity of *MKI67* and *PCNA* were used as indicators of cell proliferation in addition to BrdU staining. A significantly lower *MKI67* expression level characteristic of G_0_ was observed in the later stages of the experiment in all osteogenic conditions both in the Saos-2 cells and hMSCs (Figs. 8A and 9A). Furthermore, the decrease in *PCNA* expression after 2 and 4 days in all Saos-2 and hMSCs cultures respectively suggested the cell cycle arrest in G_0_ or exit from G_1_ and S. The reduction in transcription of both genes in all osteogenic conditions including those not containing β-Gly was possibly caused by quiescence typically arising from nutrient and/or oxygen deprivation or the build-up of toxic metabolites with increased cell numbers in longer cell cultures^28^ However, a marked decrease of BrdU-stained nuclei observed only in β-Gly-exposed Saos-2 (Fig. 4C, F) implied a unique role for β-Gly in the cell cycle arrest of Saos-2. Decreased BrdU uptake showed that fewer Saos-2 cells were in S phase after 4 days of incubation that corresponded with a reduction in proliferation. A possible cause of the latter is the cell cycle arrest triggered by terminal differentiation of Saos-2.

Terminal differentiation leads to a permanent cell cycle exit in most cell types. Terminally differentiated osteoblasts become either lining cells, osteocytes or undergo apoptosis^29^. It is necessary to highlight the mature osteoblastic phenotype of Saos-2 relative to the earlier phase of osteogenic differentiation of hMSCs as the latter affects proliferation kinetics in cells. Coelho and Fernandes^11^ reported decreased proliferation of human bone marrow cells in the presence of β-Gly after 42 days and linked it with progression of osteogenic differentiation. Interestingly in the present study, *PHEX*, an osteocyte associated marker was upregulated in osteogenically induced Saos-2 cultures, unlike hMSCs further confirming the phenotypic difference of the two in the presence of β-Gly. However, Saos-2 cells continued to express *PCNA* and *MKI67* as well as synthesising mineralised matrix which are not typical of terminally differentiated cells. Although, it is worth noting that osteocytes are notoriously difficult to maintain in vitro^30^, so terminal differentiation should not be excluded as the cause of the anti-proliferative effect of β-Gly on Saos-2.

*RUNX2* is an important regulator of osteogenic differentiation^31^ and *ALP* has often been described as an early osteogenic marker^32^. Although, since *ALP* proteins are not unique to bone, it may be argued that the *ALP* expression level is inadequate for addressing osteoblast differentiation. Saos-2 cells showed no particularly defined patterns of *RUNX2* or *ALP* expression depending on the osteogenic supplements provided and no significant difference was detected between treated and control cells. (Fig. 8C, D). This suggested that Asc, Dex and β-Gly did not affect early osteoblastic differentiation in Saos-2 cells as expected due to the relatively “mature” phenotype. This contrasted with the hMSCs where an upregulation of *RUNX2* and *ALP* expression was observed in cells subjected to Asc and/or Dex and/or β-Gly promoting early osteoblastic differentiation (Fig. 9C, D). Expression of *OCN* was increased in Saos-2 in the presence of β-Gly (Fig. 8E). Increased *OCN* transcription indicated β-Gly interaction with the later stages of osteogenesis, whilst the activity of *OCN* in hMSCs did not appear to be affected exclusively by β-Gly and was upregulated in all supplemented cultures after day 10 (Fig. 9E). This implied a transition of hMSCs into a later stage of osteogenic differentiation during the course of the experiment similarly to healthy osteoblasts described in the literature^29^. These findings suggested a potential distinct role of β-Gly in later osteogenic differentiation of Saos-2 cells not detected in hMSCs. Based on this data, it was likely β-Gly affected Saos-2 proliferation via interacting with molecular pathways in the late osteogenesis.

## Conclusion

β-Gly was shown to have a unique anti-proliferative effect on Saos-2 cells, which was not observed in a less differentiated hMSC line. Such a response may be mediated via interaction with late osteogenic differentiation of Saos-2. The derivation of Saos-2 from an osteosarcoma may have affected the response to osteogenic supplementation resulting in altered proliferation kinetics. It is important these findings are considered during selection of a model for osteogenesis in vitro.

### Supplementary Information

Supplementary Information.

## Data Availability

The data created during this research are openly available from the Open Scientific Framework repository at https://osf.io/t6skh/.
