# Supplementary material for: Differing responses of osteogenic cell lines to β-glycerophosphate
Source: Sci Rep. 2023 Sep 2;13:14472. doi: 10.1038/s41598-023-40835-w (PMC10475023; doi:10.1038/s41598-023-40835-w)
Supplement: Supplementary file 1 — Supplementary Information. [file 41598_2023_40835_MOESM1_ESM.pdf]

Differing responses of osteogenic cell lines to β-glycerophosphate.

Authors: Olga S. Yevlashevskaya[1] (oxy650@student.bham.ac.uk), BMedSc; Dr Ben A Scheven[1] (b.a.scheven@bham.ac.uk), PhD; Prof A. Damien Walmsley[1] (a.d.walmsley@bham.ac.uk), BDS, PhD;
Dr Richard M Shelton*[1] (r.m.shelton@bham.ac.uk), BDS, PhD.

Affiliations: 1. School of Dentistry, College of Medical and Dental Sciences, University of Birmingham, Birmingham, UK

Supplementary materials

Gene	Forward primer	Reverse primer
Tyrosine 3-monooxygenase, *YWHAZ*	ACTTTTGGTACATTGCTTCAA	CCGCCAGGACAAACCAGTAT
Osteocalcin, *OCN*	GGCAGCGAGGTAGTGAAGAG	CTGGAGAGGAGCAGAACTGG
Alkaline phosphatase, *ALP*	TGCTCTGCGCAGGATTG	GGAGACACCCATCCCATCTC
Runt-related transcription factor 2, *RUNX2*	CGCCTCACAAACAACCACAG	TCACTGTGCTGAAGAGGCTG
Phosphate regulating endopeptidase homolog X-linked, *PHEX*	ACTTTGCACTGCACTGGACT	TCCATCAGAAGGGCCGTAGA
Marker of proliferation Ki-67, *MKI67*	GCCCGGGGACGTAGCCTGTA	ACCGTCGACCCCGCTCCTTT
Proliferating cell nuclear antigen, *PCNA*	CCACGTCTCTTTGGTGCAG	CCGGCGCATTTTAGTATTTTGG

Table 1. Primers were designed to assess the expression of osteogenic differentiation and cell proliferation using qPCR.

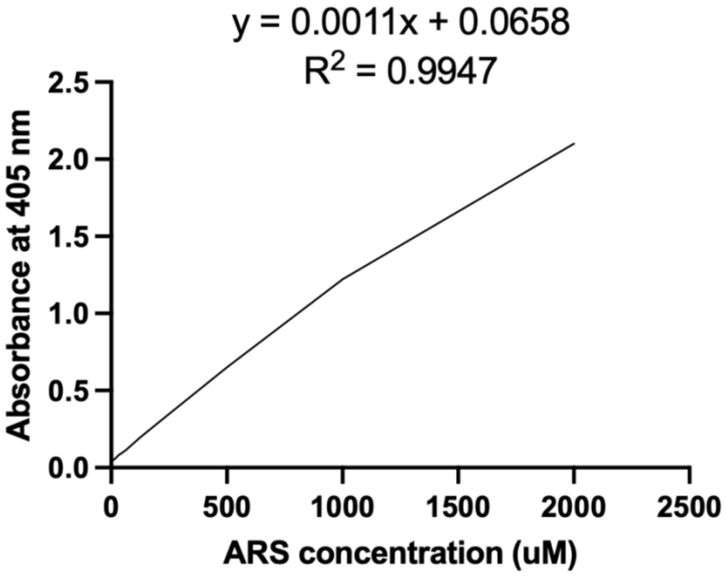

Figure 1. *Standard curve of serial dilutions of alizarin red stain used to quantify optical density of mineralising hMSCs and Saos-2 cell cultures supplemented with combinations of Asc, Dex and β-Gly.*
